# Oxytocin Ameliorates Impaired Behaviors of High Fat Diet-Induced Obese Mice

**DOI:** 10.3389/fendo.2020.00379

**Published:** 2020-07-03

**Authors:** Ryotaro Hayashi, Yoshiyuki Kasahara, Shizu Hidema, Satoshi Fukumitsu, Kiyotaka Nakagawa, Katsuhiko Nishimori

**Affiliations:** ^1^Laboratory of Molecular Biology, Graduate School of Agricultural Science, Tohoku University, Sendai, Japan; ^2^Food and Biodynamic Chemistry Laboratory, Graduate School of Agricultural Science, Tohoku University, Sendai, Japan; ^3^Nippon Flour Mills Co., Ltd., Innovation Center, Kanagawa, Japan; ^4^Department of Fetal Pathology, Graduate School of Medicine, Tohoku University, Sendai, Japan; ^5^Department of Bioregulation and Pharmacological Medicine, Fukushima Medical University School of Medicine, Fukushima, Japan; ^6^Collaborative Graduate School Program, University of Tsukuba, Tsukuba, Japan; ^7^Alliance for Research on the Mediterranean and North Africa (ARENA), University of Tsukuba, Tsukuba, Japan; ^8^Department of Obesity and Inflammation Research, Fukushima Medical University School of Medicine, Fukushima, Japan

**Keywords:** oxytocin, high-fat diet, social recognition, object recognition memory, anxiety behavior, fear-related behavior, depression behavior

## Abstract

Excessive intake of fat is a major risk factor for lifestyle-related diseases such as heart disease and also affects brain function such as object recognition memory, social recognition, anxiety behavior, and depression-like behavior. Although oxytocin (OXT) has been reported to improve object recognition, social recognition, anxiety behavior, and depression-like behavior in specific conditions, previous studies did not explore the impact of OXT in high-fat diet (HFD)-fed mice. Furthermore, it remains unclear whether intake of HFD affects OXT/oxytocin receptor (OXTR) in the brain. Here, we demonstrated that peripheral OXT administration improves not only social recognition but also object recognition and depressive-like behavior in HFD-fed mice. In contrast, peripheral OXT administration to HFD-fed male mice increased fear and anxiety-related behavior. In addition, we observed that intake of HFD decreased *OXTR* and *c-fos* mRNA expression in the hippocampus, specifically. Furthermore, peripheral OXT administration increased *OXT* mRNA expression in the hypothalamus. Altogether, these findings suggest that OXT has the potential to improve various recognition memory processes via peripheral administration but also has side effects that increase fear-related behavior in males.

## Introduction

Excessive fat intake is a major risk factor for lifestyle-related diseases such as obesity, diabetes, and metabolic syndrome. Obesity has been reported to be a risk factor for Alzheimer's disease (1, 2) and cognitive decline (3) in humans. A correlation has also been found between depression-like behavior (4), anxiety behavior (5), and obesity. In animals, previous studies have shown that intake of a high-fat diet (HFD) decreases social recognition and object recognition memory, and increases anxiety related behavior as well as depression-like behavior (6–9). Oxytocin (OXT) is a 9-amino acid neuropeptide hormone synthesized in the paraventricular nucleus (PVN) and supraoptic nucleus (SON) (10) and is secreted into blood by axons projecting axons to the posterior pituitary gland (11). Synthesized OXT has various physiological functions in a wide range of peripheral tissues, including the mammary gland, uterine smooth muscle, tongue, and bone tissue. All these tissues express oxytocin receptor (OXTR), a G protein-coupled receptor (12, 13) coupled to the q/11 type Gα subunit. The peripheral OXT/OXTR system has physiological actions such as milk ejection, parturition, and control of bone formation. OXTR expression occurs in various brain regions, including the PVN, lateral septal (LS) nucleus, hippocampus, medial amygdala (MeA), medial preoptic area (MPOA), and the bed nucleus of the stria terminalis (BNST). OXTR-expressing neurons of the PVN are exclusively glutamatergic. Moreover, OXTR expressing neurons of the BNST are GABAergic (14). OXT reaches each nucleus through the axons of oxytocinergic neurons. Moreover, it is hypothesized that OXT is released directly in these brain regions non-synaptically (15). A range of behaviors including social behaviors, and especially prosocial behaviors, are suspected to be controlled through signal regulation via the OXT/OXTR system. More specifically, pair bonding, maternal behavior, empathetic behavior, social recognition, and offensive behavior are thought to be controlled by the OXT/OXTR system (13, 16–19). OXTRs in the hippocampus regulate social recognition (20–22) and OXTRs in the amygdala controls social recognition and fear expression (23, 24). *OXT* or *OXTR* gene-deficient mice, which exhibit behavioral phenotypes such as decreased social cognitive function (25), are established as an animal model of autistic spectrum disorders (ASD). Furthermore, the OXTR is a pharmacological drug target, and human studies have shown that intranasal administration of OXT, a natural OXTR agonist, to ASD patients improved their social communication and social reciprocity (26, 27). Amelioration in social cognitive function was also observed in a valproic acid (VPA)-treated ASD mouse model, upon administration of intranasal OXT (28).

The effects caused by peripheral administration of OXT on social recognition and object recognition are unclear. In previous studies, the effect of peripherally administrated OXT on cognitive function varied among animal models. For example, intranasal administration of OXT to VPA-treated mice restored social cognitive function but did not improve objective cognitive function (28). In addition, it has been reported that intracerebroventricular (i.c.v.) administration of OXT to *Shank3*-knockout (KO) mice can restore their social cognitive function (29), and nasal administration of OXT to tail shock stress-treated rats can also restore their cognitive function (30). Peripheral administration of OXT has antidepressant effects (31), and has also been shown to exert the opposite effects on anxiety behavior. OXT may act on the PVN, and OXT administration to PVN and ventricle exhibit anxiolytic effects (32, 33). However, anxiogenic effects have been caused by repeated OXT i.c.v. administration (34) and this may be related to the septal OXTRs (35). In addition, because OXT KO mice and OXTR KO mice show significantly increased body weight (16, 36), the OXT/OXTR system appears to be strongly involved in obesity and/or control of food intake (37, 38). As OXT appears to have a dual function in body weight control and recognition, we speculated that OXT is more effective for ameliorating the recognition impairment caused by a HFD.

However, the effects of peripherally administrated OXT on social cognition, object recognition, anxiety, and depression-like behavior in mice on a HFD have not been reported to date. The aim of this study was thus to evaluate the effects of peripherally administrated OXT on the various cognitive functions—social recognition, object recognition, anxiety behavior, and depression-like behavior—in HFD-fed mice. In addition, by inferring the relationship between HFD intake and the OXT/OXTR system in the brain, we attempted to elucidate the mechanisms underlying the cognitive function deficits that we observed in HFD-fed mice.

## Materials and Methods

### Animals and Diets

**Figure 1:**
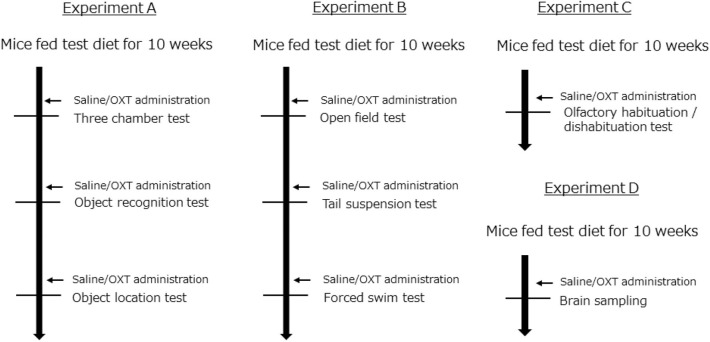
Experimental design. All experiments (A–D) were performed independently. In each experiment, mice were randomly distributed into the following groups: (1) a group fed a normal diet (ND); (2) a group fed a high-fat diet (HFD); and (3) a group fed a high-fat diet and also administrated oxytocin (HFD + OXT) (Experiment A: *n* = 21, B: *n* = 10, C: *n* = 8, D: *n* = 15 mice per group).

**Figure 2:**
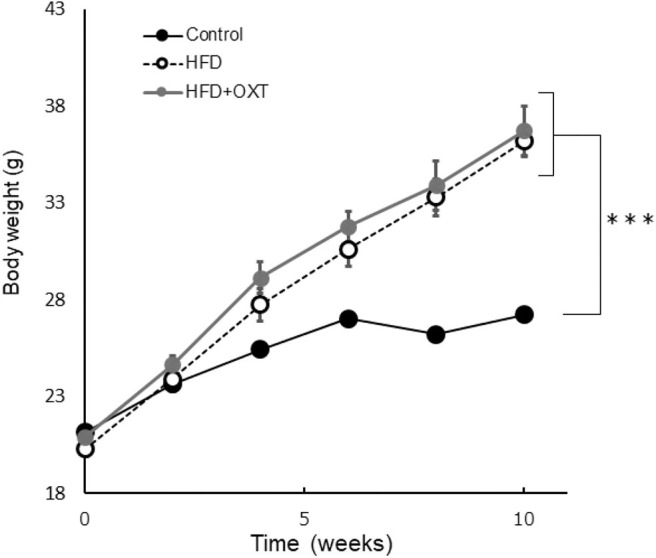
Effect of high-fat diet to body. The body weight of each groups before behavioral testing and OXT administration (*n* = 8 mice per group). Results are displayed as mean ± SEM, ***p < 0.005.

C57BL/6J male mice were obtained from Japan SLC (Hamamatsu, Japan). Forty-nine mice per group were housed in individual cages with a 12-h light-dark cycle. The mice were randomly divided into three groups (ND: normal diet; HFD: high-fat diet; and HFD + OXT: high-fat diet + oxytocin). The ND group was fed normal diet (D12450J; Research diet, New Brunswick, NJ). Both the HFD and HFD + OXT groups were fed HFD (D12492; Research diet, New Brunswick, NJ). The mice were fed the test diet starting at the age of 6 weeks for a duration of 10 weeks before the behavioral tests were initiated. All groups had *ad libitum* access to food and water. Altogether, we performed four independent experiments involving all three groups of mice (Experiment A–D; Figure 1). The body weight of the mice was measured once every 2 weeks until the behavioral testing (Figure 2). All animal experiments were approved by the Institutional Animal Care and Use Committee of Tohoku University and the Animal Experiment Committee of Nippon Flour Mills.

### Drug Administration

OXT was purchased from Peptide Institute Inc. (Osaka, Japan) and dissolved in 0.9% NaCl solution before use. OXT (1 mg/kg) or saline was intraperitoneally administrated at a volume of 10 ml/kg body weight, 45 min before each behavioral test (Figure 1). We administrated saline to the ND and HFD group, and OXT to the HFD + OXT group. We used the same dose (1 mg/kg) of OXT as in previous studies (7, 39, 40).

### Behavioral Tests

The battery of behavioral tests began after intakes of the test diet for 10 weeks (Figure 1). Thirty-nine mice per group were used for the behavioral testing. The interval between each behavioral test was 1–7 days. The mice were placed in a test room for 1 h before starting the test. After each behavioral test, we returned the mice to their individual cage. We recorded and analyzed the results of some behavioral tests using the video-tracking ANY-maze software (Stoelting Co., Wood dale, IL, USA).

### Three-Chamber Sociability and Social Novelty Test (TCT)

**Figure 3:**
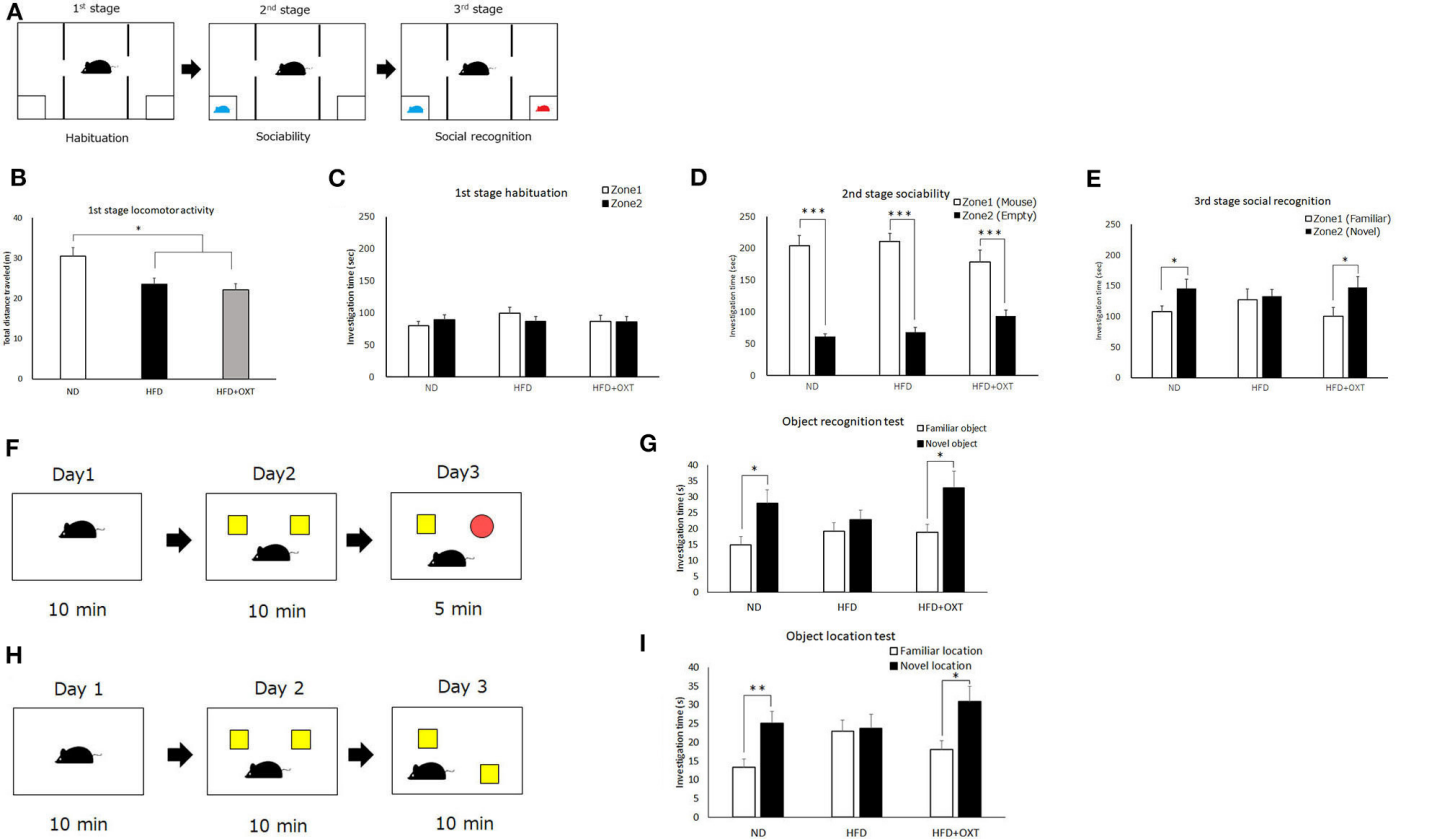
Effect of high-fat diet and oxytocin administration on social and object recognition memory. Schematic illustration of the three-chamber test: first stage (habituation); second stage (social interaction) and third stage (social recognition) **(A)**. **(B–E)** Time spent in each zone during 10 min: social interaction **(D)** and social recognition **(E)** (*n* = 21 mice per group). Schematic illustration of object recognition test **(F)**. Time to explore object in day 3 (*n* = 13 mice per group) **(G)**. Schematic illustration of object location test **(H)**. Time to explore object in day 3 (**I**; *n* = 10 mice per group). Results are displayed as mean ± SEM, *p < 0.05, **p < 0.01, ***p < 0.005.

The three-chamber test was carried out to evaluate mice sociability and social recognition according to our previous reports (41, 42). Social approach was assessed in a three-chamber box (41 × 21 × 35 cm) under 10 LX LED lights using a test procedure that consisted of three stages. In the first stage, the test mice were placed in the box and left on their own for 10 min so they would habituate to their new environment. In the second stage, an unfamiliar male stimulus mouse that had never contacted the test mice. was placed in a triangle mesh cup at one corner of the box and the test mice were allowed to freely move around the chambers and investigate the newcomer for 10 min (sociability test). In the third stage, a novel stimulus mouse was located in another cup at the opposite corner, and again the test mice were allowed to freely move around between the chambers and investigate the two stimulus mice for another 10 min (social recognition; Figure 3A). All tests were recorded and the stimulus mice investigation time, which was defined as the amount of time that the test mice were located within 5 cm of each cup, was analyzed using the ANY-maze software.

### Object Recognition Test (ORT)

The three-stage object recognition test (ORT) was performed as previously described (43), with slight modifications. Briefly, in the first stage, mice were placed into a square open field box (40 × 40 × 40 cm) under 47 LX LED lights for 10 min and allowed to explore freely prior to being returned to their home cages. After 24 h, in the second stage, two identical objects were placed at two different corners of the field box, and the mice were placed into field and allowed to explore the two objects freely for 10 min, prior to returning to their home cage. After another 24 h, in the third stage, the mice were returned to the field box and allowed to explore one of the original objects together with a novel object (Figure 3F). The exploration time with the familiar object and the novel object was measured.

### Object Location Test (OLT)

The object location test (OLT) was performed as previously described (43), with slight modifications. Briefly, the three-stage procedure was performed in a square open field (40 × 40 × 40 cm) with a black pattern picture on the wall under 47 LX LED lights. In the first stage, mice were placed into the open field box for 10 min and allowed to explore freely and prior to being returned to their home cage. After 24 h, in the second stage, two identical objects were placed at two different corners of the field. The mice were allowed to explore the two objects freely for 10 min and then returned to their home cage. After another 24 h, in the third stage, one of the objects was placed in a different corner and the mice were allowed to freely explore the originally and newly placed objects (Figure 3H). The exploration time with each object was measured.

### Olfactory Habituation/Dishabituation Test

The olfactory habituation/dishabituation test was carried out as previously described (8, 44). The odorant stimuli used were tap water, vanilla and lemon extracts (two non-social odorant stimuli), and social odorants prepared by wiping cotton tips across the bed of different male mice. The stimuli-soaked cotton swabs were presented for 3 min and then replaced with a fresh swab scented with the same odorant for a total of three presentations, with a 2 min-interval between them. We recorded the cumulative time that mice spent sniffing the cotton swabs.

### Open Field Test

The open field test was performed in a 50 × 50 × 40 cm opaque gray chamber under 450 LX LED lights as previously described (45). After a 10-min acclimation period in the test arena, the mice were allowed to freely explore the field for 10 min. Their movements and other data were recorded and analyzed using the ANY-maze software. The chamber was divided into 100 squares by the software, with 16 squares covering the center zone of the test arena, and the remaining 84 squares being regarded as the corner zone. The total time spent and the duration of freezing in each zone were analyzed.

### Tail Suspension Test

The tail suspension test was performed to evaluate the susceptibility to depression-like states, as previously described (46). Each mouse was suspended 20 cm above the ground by the tail for 6 min using tape. The experiment was recorded using a video camera, and the last 3 min were used for analysis. The amount of time that the mice spent being immobile was recorded manually by an experimenter blinded to the mouse group.

### Forced Swim Test

The forced swim test was performed to evaluate depressive-like behavior, as previously described (47). Each mouse was individually placed in an open, clear Plexiglas cylindrical container (40 cm tall x 20 cm diameter) containing 20 cm of water at 24°C ± 1°C for 6 min. The first 3 min were used for habituation time and the last 3 min for analysis. The experiment was recorded by video camera. The immobility time was recorded manually by an experimenter blinded to the mouse group.

### Sacrifice and Tissue Harvesting

We sacrificed the mice 105 min after OXT administration (Figure 1, Experiment D). Brain tissue samples were collected from the social recognition-related brain areas, including the prefrontal cortex, lateral septum, medial amygdala, hippocampus and the OXT-producing area of the brain (hypothalamus). Blood samples were also collected. The samples were allowed to clot in tubes and centrifuged at 4°C for 10 min at 3,000 rpm to separate out the serum. The serum fraction and brain samples were stored at −80°C for further analysis.

### RNA Isolation and Real-Time Polymerase Chain Reaction (PCR)

Total RNA was isolated from brain tissues using a RNeasy Lipid Tissue Mini Kit (Qiagen, Hilden, Germany) and reverse transcription (RT) was performed using a PrimeScript RT reagent Kit with gDNA Eraser (Takara, Kyoto, Japan). Real-time quantitative PCR was then performed using the Thermal Cycler Dice Real Time System III (Takara, Kyoto, Japan). For each sample, a parallel reaction was set up with b-actin. Each reaction was performed in duplicate. In real-time PCR analysis, the following primers were used: OXTR(NM_001081147): forward(GGAGCGTCTGGGACGTCAAT), reverse(AGGAAGCGCTGCACGAGTT); OXT(XM_006498910): forward(TGGCTTACTGGCTCTGACCT), reverse(AGGCAGGTAGTTCTCCTCCTG); β-actin(NM_007393): forward(AGCCTTCCTTCTTGGGTA), reverse(GAGCAATGATCTTGATCTTC); c-fos(NM_010234): forward(CAAAGTAGAGCAGCTATCTCC), reverse(CTCATCTTCAAGTTGATCTGT).

### Immunohistochemistry

Mice were transcardially perfused with 10 mM PBS, followed by 4% PFA in PBS 135 min after OXT administration (Figure 1, Experiment D). Brains were post-fixed in 4% PFA overnight at 4°C and cyro-protected for 72 h in 30% sucrose at 4°C before freezing in OTC compound. Coronal sections at 30 μm intervals were processed using a cryostat (Leica Biosystems, Nussloch, Germany). Sections were stored in PBS. Floating sections were used to perform immunohistochemical labeling for detection of OXT. Briefly, sections were washed in PBS for 10 min, incubated for 30 min at room temperature in PBS containing 0.3% Triton X-100, washed in PBS for 10 min, incubated in PBS containing 5% MeOH, 0.2% Triton X-100 and 1.5% H_2_O_2_ for 60 min in order to inactivate endogenous peroxidase of sections. Sections were washed in PBS for 10 min, blocked in PBS containing 0.3% TritonX-100 and 10% normal horse serum for 30 min at room temperature.

Sections for immunocytochemical detection of OXT were incubated with rabbit anti-OXT antibody (diluted 1:1000; ImmunoStar, Hudson, WI, USA) at 4°C for 24 h. Subsequently, sections were washed three times in PBS and incubated with peroxidase-labeled secondary antibodies (goat anti-rabbit IgG; diluted 1:500; G-21234; Thermo Fisher Scientific, Waltham, MA, USA) at 4°C for 24 h. OXT immunoreactivity was visualized as brown cytoplasmic precipitate with DAB (DAB Peroxidase Substrate Kit; SK-4105; Vector Laboratories, Burlingame, CA, USA). Sections were observed with a microscope (BZ-X700; Keyence Co. Ltd., Osaka, Japan). The number of OXT positive cells were counted manually by an experimenter blinded to the mouse group, and taken as the average of the three slides from each mouse.

### Measurement of Serum OXT

We extracted OXT from blood serum using C-18 column according to the manufacturer's protocol. Extracted OXT levels were measured using an enzyme-linked immunosorbent assay (ELISA) kit (intra-assay coefficients of variation <13.3%, inter-assay coefficients of variation <20.9%, Enzo Life Sciences, Inc., Farmingdale, NY, USA).

### Statistical Analysis

All values are expressed as means ± standard error of the mean (SEM). Data from the three-chamber, object recognition, and object location tests were analyzed by Welch's *t*-test. Other experimental data on multi-group comparisons were first analyzed by Levene's test for variances. We performed one-way ANOVA and *post-hoc* Tukey-Kramer tests as parametric analysis or Kruskal-Wallis test and *post-hoc* Steel-Dwass test as non-parametric analysis. *P* velue <0.05 were considered statistically significant. Statistical analyses of the experimental data were performed with SPSS (IBM Corp., Armonk, NY, USA) and R software.

## Results

### Effect of OXT Administration on Social and Object Recognition Memory in HFD-Fed Mice

We analyzed recognition memory by using the three-chamber, object recognition, and object location tests. Firstly, we performed the three-chamber test to assess sociability and social recognition (Figure 3A). In the first habituation stage, locomotor activity of HFD and HFD + OXT mice was decreased compared to ND mice [ANOVA: F_(2,60)_ = 7.99, *p* = 0.001; Tukey's test: ND vs. HFD, *p* = 0.012; ND vs. HFD + OXT, *p* = 0.001; Figure 3B]. However, the ND, HFD, and HFD + OXT mice all stayed in two zones for similar amounts of time (ND: *p* = 0.37; HFD: *p* = 0.29; and HFD + OXT: *p* = 0.95; Figure 3C). In the second stage, the ND, HFD, and OXT mice all spent more time with the stimulus mice than with the empty cup (ND: *p* < 0.005; HFD: *p* < 0.005; and HFD + OXT: *p* < 0.005; Figure 3D). In the third stage, the ND mice spent more time with the novel mice than the familiar ones, whereas the HFD mice spent a similar length of time with the novel and familiar mice. The OXT mice spent more time with the novel mice than the familiar one (ND: *p* = 0.042; HFD: *p* = 0.79; and HFD + OXT: *p* = 0.048; Figure 3E). These results indicate that the intake of a HFD impaired the ability of mice in social recognition but not their sociability, and that OXT administration ameliorated their impaired social recognition.

In the object recognition test (Figure 3F), the ND mice investigated the novel object for a longer time than the familiar object, whereas the HFD mice investigated the novel and familiar objects for a similar amount of time. The OXT-administrated mice investigated the novel object for longer than the familiar one (ND: *p* = 0.016; HFD: *p* = 0.39; and HFD + OXT: *p* = 0.031; Figure 3G). These results indicated that a HFD intake impaired object recognition memory in mice, and that OXT administration ameliorated this.

In the object location test (Figure 3H), the ND mice spent a longer time investigating the novel location of the object than the familiar object location, whereas the HFD mice spent a similar length of time investigating the objects in the novel and familiar location. OXT-administrated mice investigated the novel location object for a longer time than the familiar location object (ND: *p* = 0.006; HFD: *p* = 0.86; and HFD + OXT: *p* = 0.018; Figure 3I). These results indicated that mice on a HFD had impaired object location memory, and that OXT administration ameliorated their this.

### Anxiety- and Fear-Related Behavior in HFD-Fed Mice, and the Effect of OXT

**Figure 4:**
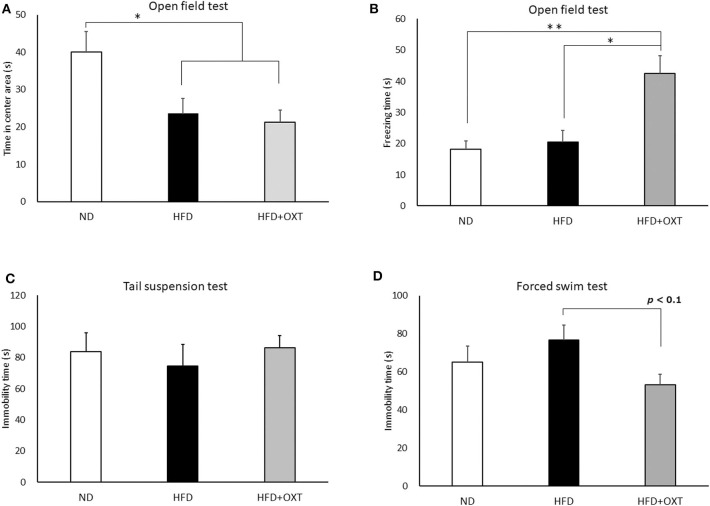
Effect of high-fat diet and oxytocin administration on anxiety behavior, fear-related behavior, and depressive-like behavior. Effect of a high-fat diet and oxytocin administration on the time spent in the center area **(A)** and on freezing behavior **(B)** in the open field (*n* = 9–10 mice per group). Effect of a high-fat diet and oxytocin administration on the immobility time in the tail suspension test **(C)** and forced swim test (**D**; *n* = 9–10 mice per group). Results are displayed as mean ± SEM, *p < 0.05, **p < 0.01.

We analyzed anxiety- and fear-related behavior by using the open field test. The time spent in the center area by the HFD and the HFD + OXT mice was reduced compared to that of the ND mice (ANOVA: F_(2,26)_ = 5.45, *p* = 0.011; Tukey's test: ND vs. HFD, *p* = 0.039; ND vs. HFD + OXT, *p* = 0.014). The HFD and the HFD + OXT mice spent similarly shorter times in the center area (Tukey's test: *p* = 0.931; Figure 4A). The freezing time in the whole area was similar between the ND and the HFD mice (Levene's test: *p* = 0.024; Kruskal-Wallis test: *p* = 0.009; and Steel-Dwass test: *p* = 0.84). In contrast, the HFD + OXT mice showed more freezing time compared to the ND and HFD mice (Steel-Dwass test: ND vs. HFD + OXT, *p* = 0.0017; HFD vs. HFD + OXT, *p* = 0.038; Figure 4B).

### Effect of HFD and OXT Administration on Depressive-Like Behavior

We analyzed depressive-like behavior by using the tail suspension and forced swim tests. In the tail suspension test, immobility time was similar among the ND, HFD, and HFD + OXT mice (ANOVA: F_(2,26)_ = 0.282, *p* = 0.757; Figure 4C). In contrast, in the forced swim test, the immobility time of the OXT mice tended to be shorter than that of the HFD mice (ANOVA: F_(2,26)_ = 2.39, *p* = 0.112; and Tukey test: *p* = 0.093). In contrast, the ND and HFD mice showed similar immobility times (Tukey test: *p* = 0.511; Figure 4D).

### Effect of HFD and OXT Administration on Sensory Function

**Figure 5:**
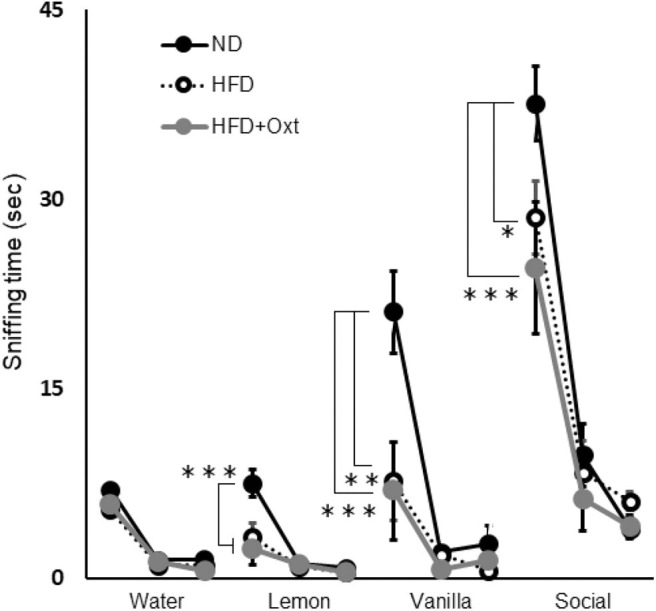
Effect of high-fat diet and oxytocin administration on sensory functions. Sniffing time for each odor in the olfactory habituation/dishabituation test (*n* = 8 mice per group). Results are displayed as mean ± SEM, *p < 0.05, **p < 0.01, ***p < 0.005.

We performed the olfactory habituation/dishabituation test to examine olfaction in HFD-fed mice by using the mouse's tendency to investigate a novel odor for a longer time. The amount of time spent sniffing the first swab dipped into water was similar among all three groups [ANOVA: F_(2,21)_ = 1.29, *p* = 0.296; Figure 5]. However, when lemon, vanilla, and social odors were used, the first sniffing time of HFD mice decreased compared to that of ND mice ([lemon, ANOVA: F_(2,21)_ = 13.3, *p* < 0.001; and Tukey test: *p* = 0.002], vanilla [ANOVA: F_(2,21)_ = 8.43, *p* = 0.002; and Tukey test: *p* = 0.006], and social odor [ANOVA: F_(2,21)_ = 8.16, *p* = 0.002; and Tukey test: *p* = 0.032]). Furthermore, the sniffing time for these substances in the HFD + OXT mice was almost equivalent to that in the HFD mice (Tukey test: lemon, *p* = 0.682; vanilla, *p* = 0.979; and social odor *p* = 0.462). In HFD-fed mice, the impaired sensory function was not ameliorated by peripheral OXT administration.

### Effect of HFD and OXT Administration on mRNA Expression of *OXTR* and *c-fos* Genes

**Figure 6:**
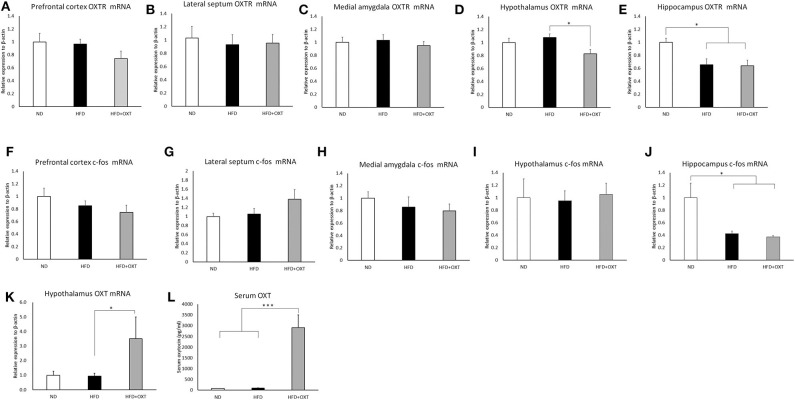
Effect of high-fat diet and oxytocin administration on mRNA expression and serum OXT. Expression of *c-fos, OXTR*, and *OXT* mRNA was measured by real-time PCR (*n* = 6–10 mice per group). *c-fos* mRNA expression in social recognition-related brain areas (prefrontal cortex, lateral septum, medial amygdala, hypothalamus, and hippocampus) **(A–E)**. *OXTR* mRNA expression in the above-mentioned brain regions **(F–J)**. *OXT* mRNA expression in the hypothalamus **(K)**. Serum oxytocin **(L)**. Results are displayed as mean ± SEM, *p < 0.05, ***p < 0.005.

We analyzed brain *OXTR* and *c-fos* gene mRNA expression in the social recognition-related nuclei including the prefrontal cortex, lateral septum, medial amygdala, hypothalamus, and hippocampus. In the prefrontal cortex [ANOVA: F_(2,20)_ = 1.49, *p* = 0.24], lateral septum [ANOVA: F_(2,19)_ = 1.14, *p* = 0.893] and medial amygdala [ANOVA: F_(2,19)_ = 3.17, *p* = 0.732], a HFD and the subsequent and peripheral administration of OXT did not influence the expression of *OXTR* (Figures 6A–C). In the hypothalamus, *OXTR* mRNA expression in the OXT-administrated mice was decreased compared to the HFD mice (ANOVA: F_(2,21)_ = 5.45, *p* = 0.12; and Tukey test: *p* = 0.01; Figure 6D). In the hippocampus, *OXTR* mRNA expression in the HFD mice and OXT-administrated mice was decreased compared to the ND mice [ANOVA: F_(2,24)_ = 6.40, *p* = 0.006; and Tukey test: *p* = 0.015, *p* = 0.011; Figure 6E]. In contrast, intake of HFD and peripheral OXT administration did not affect *c-fos* gene expression in the prefrontal cortex (ANOVA: F_(2,20)_ = 1.83, *p* = 0.18), lateral septum (ANOVA: F_(2,24)_ = 1.98, *p* = 0.159), medial amygdala [ANOVA: F_(2,22)_ = 5.57, *p* = 0.557] and hypothalamus [ANOVA: F_(2,20)_ = 0.66, *p* = 0.936; Figures 6F–I]. In the hippocampus, *c-fos* mRNA expression in the HFD and OXT-administrated mice was decreased compared to the ND mice [ANOVA: F_(2,23)_ = 7.62, *p* = 0.003; and Tukey test: *p* = 0.017, *p* = 0.003; Figure 6J].

### Effect of HFD and OXT Administration on mRNA Expression of *OXT* Gene

We analyzed mRNA expression from the *OXT* gene in the hypothalamus, which is the area of the brain that predominantly synthesizes OXT. In the hypothalamus, the HFD did not influence *OXT* gene expression. In contrast, in the OXT-administrated mice, *OXT* gene expression was increased, compared to that in the HFD mice [ANOVA: F_(2,24)_ = 3.87, *p* = 0.035; and Tukey test: *p* = 0.049; Figure 6K]. Peripheral OXT administration also increased *OXT* gene expression in HFD-fed mice.

### Effect of HFD and OXT Administration on Serum OXT and Body Weight

The body weight of HFD-fed mice (HFD, HFD+OXT mice) was increased compared to normal diet fed mice (Kruskal-Wallis test: *p* < 0.001; and Steel-Dwass test *p* < 0.001, *p* < 0.001). We analyzed serum OXT after administration of OXT or saline. In the OXT-administrated mice, the level of serum OXT was increased compared to that of the ND and HFD mice (Kruskal-Wallis test: *p* < 0.01; and Steel-Dwass test *p* < 0.001, *p* < 0.001; Figure 6L).

### Effect of HFD and OXT Administration on OXT Expression in the PVN

**Figure 7:**
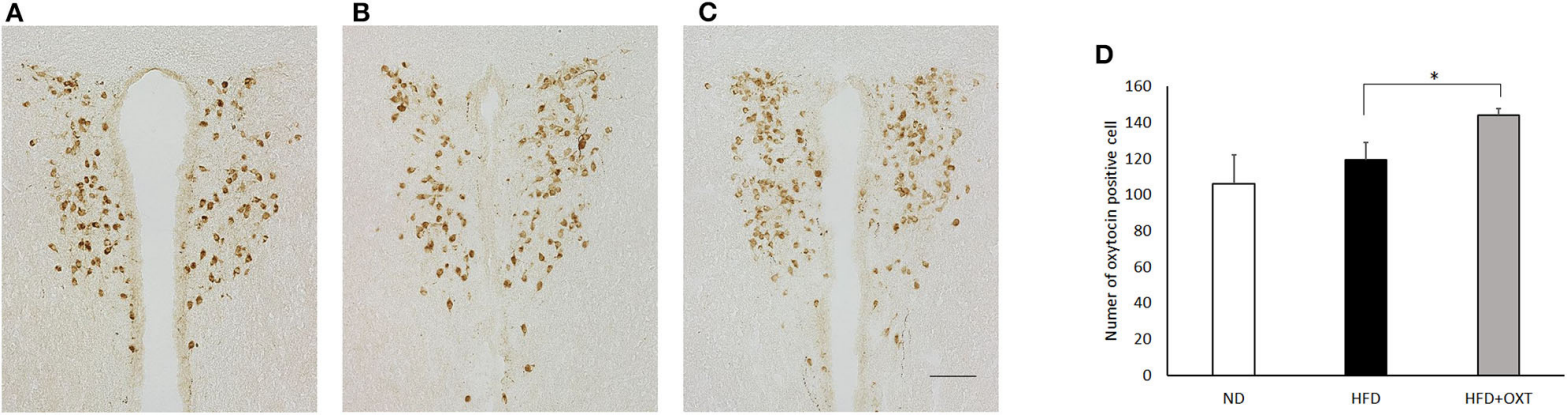
Effect of high-fat diet and oxytocin administration on OXT expression in the PVN. Expression of OXT protein in the PVN was measured by immunohistochemistry. Photographs showing OXT immunoreactivity (brown cell body profiles) in the PVN (**A**:ND, **B**:HFD, **C**:HFD+OXT mice). Number of OXT expressing cells after IP injection of OXT in PVN (*n* = 4–5 mice per group) **(D)**. Scale bar: 100 μm. Results are displayed as mean ± SEM, *p < 0.05.

We analyzed OXT expression in the PVN by immunohistochemistry method (Figures 7A:ND, 7B:HFD, 7C:HFD+OXT mice). In the OXT-administrated mice, the number of OXT expressing cells in the PVN was increased compared to that in the HFD-fed mice (Kruskal-Wallis test: *p* < 0.05; and Steel-Dwass test *p* < 0.05) (Figure 7D).

## Discussion

Obesity induced by a HFD is recognized as one of the major risk factors for lifestyle-related diseases, but it may also affect psychopathology in patients and lead to abnormal behaviors in their social life. In particular, obesity is associated with Alzheimer's disease (1, 2), cognitive decline (3), depression-like behaviors (4), and anxiety behaviors (5). Therefore, a comprehensive treatment approach is required to correct the behavioral abnormalities caused by obesity.

OXT is known to improve social recognition. On the other hand, OXT has also been reported to suppress food intake (48) and fat accumulation (49). However, to date, the effect of OXT administration on behavioral abnormalities induced by a HFD, including overall cognitive functions such as social cognition, object cognition, anxiety behavior, fear-related behavior, and depression-like behavior remain inconclusive.

In this study, we demonstrate that the peripheral administration of OXT in HFD-fed mice ameliorates the decline in social recognition, object recognition and object location memory. On the other hand, we found that anxiety-related behavior was increased by peripheral OXT administration in HFD-fed mice.

OXT can have controversial effects, depending on the dose and administration route. For example, OXT had anxiogenic effects when dosed at 10 ng/h for 15 days via i.c.v. administration (34) and anxiolytic effects when dosed at 10 mg/kg via intraperitoneal (i.p.) administration (50). The purpose of this study was to examine the effect of OXT on behavior in relation to social and non-social cognitive impairment in HFD-fed mice. The administration dose (1 mg/kg, i.p.) was based on that used in a previous study (6).

Intake of a HFD decreased locomotor activity. However, in the first stage of the three chamber test, the search time for the two corners was similar between the groups, suggesting that a decrease in search activity does not affect object identification. In the first stage of the three chamber test, there was no change in the search activity after the administration of OXT, suggesting that OXT restored cognitive function independently of the activity level.

With regards to recognition memory, OXT has a consistent effect with different administration routes. Intranasal administration of OXT ameliorated social recognition but not object recognition in a valproic acid (VPA)-treated ASD model in mice (28). Intraventricular OXT administration ameliorated social recognition in shank3 KO mice. Moreover, nasal OXT administration improves object recognition memory in stressed rats (30).

Behavioral abnormalities similar to previous studies were observed in HFD-fed mice, including object recognition, object location memory, anxiety behavior, and depressive behavior. In addition, we found that social recognition was decreased in HFD-fed mice. A few reports exist on the social cognitive function of HFD-fed mice. One study investigating mice on a HFD demonstrated a decrease in social recognition memory (8), while another study reported that their social memory did not decrease (51). These contradictory results may be due to differences in the protocol design for the behavioral testing. In the present study, we confirmed a reduction in social memory in HFD-fed mice, consistent with previously reported findings (8).

Social communication is a fundamental ability of social animals, whereas impairment of communication ability is observed as a core symptom in ASD patients and is an important diagnostic criterion. Also, impairment of social memory is observed as a common feature in ASD animal models such as *OXTR* KO mice, Shank3 KO mice, and mice that receive fetal VPA administration; these models are widely used to explore the mechanisms underlying the development of ASD in the search for new therapeutic strategies.

On the other hand, there are few animal models in which social memory deteriorates due to environmental factors after birth. The HFD-fed mice model showed a decline in social memory due to environmental factors after birth, therefore it could reflect some aspects of the symptoms of ASD in a way different to other animal models of ASD. However, the detailed mechanisms are still unclear and further studies are required. It is expected that HFD-fed mice could be used to study the mechanisms underlying psychiatric diseases accompanied by social cognitive dysfunction such as dementia, or impaired social memory such as ASD. Moreover, HFD-fed mice have the potential to be utilized as a new psychiatric disease animal model for the development of new medicines to treat or even cure these disorders.

Some studies have reported a potential or direct relationship between of the OXT/OXTR signaling system and social recognition, object recognition, object location memory, anxiety behavior, fear-related behavior, and depressive behavior (13, 35, 52, 53). We hypothesized that the OXT / OXTR system is involved in the mechanisms leading to behavioral abnormalities in HFD-fed mice. Therefore, we evaluated the mRNA expression of *OXT, OXTR*, and *c-fos* in the brain of mice fed with a HFD. The mRNA levels of *OXTR* and *c-fos* genes were decreased in the hippocampus of HFD-fed mice. As we carried out this experiment by injection of OXT or saline solution intraperitoneally, the level of resultant c-fos expression may be partially due to an effect caused by handling of the mice and administration of these solutions.

Previous studies have reported that the hippocampus has a critical role in social and emotional behaviors, such as social recognition (22, 54), anxiety behavior, fear-related behavior (55), and depressive behavior (56). OXTR-expressing neurons in the hippocampus were also reported to play an essential role in social memory (20). These reports suggested a close relationship between the OXT/OXTR signaling system and impaired behaviors in HFD-fed mice.

Serum OXT levels have been shown to decrease in HFD mice (57). One possible explanation for the impairment in multiple types of behavior among HFD-fed mice could be a reduction in OXT/OXTR signaling. Based on this hypothesis, we attempted to cure impaired behaviors in HFD-fed mice through the peripheral administration of OXT. We found that peripheral administration of OXT could ameliorate some of the impaired behaviors in HFD-fed mice. Also, peripherally administrated OXT has been reported to activate OXT neurons in the PVN via the peripheral vagus nerve (58) and lead to increased release of internal OXT, which is synthesized in the PVN (59). On the other hand, it has been reported that peripherally administrated OXT can reach the brain in rhesus macaque monkeys (60). It has been reported that intake of a HFD causes blood-brain barrier (BBB) dysfunction and increases BBB permeability (61). We could not exclude the possibility that OXT permeability and brain translocation are both increased in mice fed on a HFD.

In our study, peripheral OXT administration in HFD-fed mice restored social cognitive function, object cognitive function, object location memory, and depression-like behavior. A similar effect has been observed on impaired social recognition in HFD-fed rats (7). Thus, we suspected that may be a common mechanism inducing social memory in mammals on a HFD. In a similar experiment involving the intranasal administration of OXT in a mouse model of ASD, where the ASD was induced by exposure to valproic acid *in utero*, restoration of cognitive function arose in a different manner, showing that the underlying mechanisms depend on the animal model as well as the route of OXT administration (28).

Object recognition is not affected by the administration of i.c.v. OXTR antagonists to male mice, OXT knockout mice showed no change in spatial memory performance (19), and hippocampal OXTR was not necessary for object recognition (20). As a result, the OXT / OXTR system in the brain is thought to play a minor role in non-social recognition (15).

On the other hand, chronic i.c.v. administration of OXT slightly improves object recognition in male rats for 7 days (62), and nasal OXT improves cognitive function in stressed rats (30). It is possible that the administration of OXT by multiple administration routes could contribute to an improvement in object recognition. However, since nasal OXT administration did not improve object recognition in VPA-treated mice (28), further studies are needed on animal models and routes of administration to evaluate the effects of OXT on cognitive function.

With regard to social recognition, OXTR KO mice have impaired social cognitive function, and hippocampal OXTRs (20) are essential for social cognitive function. Therefore, it can be said that the OXT/OXTR system is essential for social cognitive functions.

Improvement of social cognitive function through OXT administration has been confirmed in male rats by intraventricular administration (63), intranasal administration (28), and intraperitoneal administration (7). Therefore, it was considered that the administration of OXT by multiple administration routes may contribute to improved social cognitive function.

The hippocampus is one of the brain areas that regulate cognitive functions. Previous studies have suggested that the OXT/OXTR system is involved in hippocampal neurogenesis (64) and the subsequent formation of hippocampal-dependent learning and memory (65). Thus, one possible mechanism underlying the restoration of cognitive functions in HFD-fed mice following administration of OXT may involve neurons expressing *OXTR* in the hippocampus. In the near future, we would thus like to confirm the effects of peripherally administrated OXT on the activation of hippocampal neurons expressing *OXTR* in OXTR-EYFP (OXTR-Venus) knock-in mice fed on a HFD, in which OXTR neurons express Venus protein (33).

The peripheral administration of OXT to HFD-fed mice did not affect the duration of time spent in the center in our open field tests. Previous studies however reported that wild-type rats fed with normal chaw showed reduced anxiety behavior when subjected to the open field test following the peripheral administration of OXT (66). In our study, anxiety behavior was not reduced in mice, suggesting that the pathology induced by a HFD and the physiological effects of OXT administration may not always be the same in rats and mice.

Freezing time is one of the indices used to indicate the level of fear. In the present study, we showed that freezing time was increased when OXT was administrated to HFD-fed mice. This finding supports the notion that OXT may exert opposite functions in enhancing or reducing anxiety and fear in male rats and male humans, depending on the context, as found in previous studies (35, 67). The effect of OXT on anxiety-related behavior in this study was not observed in the center time of the open field test, and only freezing behavior induced anxiety. In the four plate test, the i.p. administration of 3 mg/kg OXT had no effect, but 10 mg/kg OXT had an anxiolytic effect (50). It is thought that the type of behavioral test and dose of oxytocin could alter the effects of oxytocin on anxiety-related behavior. Further studies are necessary to examine the dose and administration route of OXT and their effects on fear behavior in HFD-fed mice.

Previous studies using the tail suspension and forced swim tests to evaluate the depression-like behavior of HFD-fed mice yielded inconsistent results, with some studies describing an increase in depression-like behavior (68), while others did not show an increase (8). The index of depression-like behavior might differ depending on experimental factors like the behavioral test battery and environment. In the forced swim test, we detected a tendency of OXT to have an anti-depressant effect when administrated to HFD-fed mice. This result is consistent with an antidepressant effect of OXT (31).

Next, we evaluated olfactory function, on the basis of the ability of mice to identify individual mice which they had encountered before (69). In a previous study (8), mice fed a HFD showed a decrease in olfaction for physical odors, but in the present study, we found that olfaction for social odors in HFD-fed mice was also decreased. On the other hand, peripherally administrated OXT did not restore impaired olfaction for both physical and social odors in HFD-fed mice. These results imply that OXT might restore the impaired social memory of HFD-fed mice by a mechanism other than olfactory function.

As revealed by anatomical analyses, peripherally administrated OXT reduced the expression of *OXTR* mRNA in the hypothalamus of HFD-fed mice. A previous study reported that chronic lateral ventricular infusion of OXT at 10 ng/h for 15 days decreased mRNA expression of OXTR in dorsolateral septum (DLS), ventrolateral septum (VLS), basolateral amygdala, medial amygdala, nucleus of the amygdala (CeA), and median raphe nucleus and increased anxiety behavior (34).

Chronic third ventricular infusions of OXT caused down-regulation of Arc and VMH OXTRs in rats (70). Repeated administration of OXT to the lateral ventricle decreased OXTR binding in the VMH, anterior olfactory nucleus (AOP), BNST, CeA and the ventral septum (VS) (71). Repeated administration of OXT (1,000 ng) did not change OXTR binding in AOP, BNST, amygdala, VMH, and vs. after 24 h. However, there was a slight decrease in OXTR binding in the AOP 72 h after administration (71).

These studies suggest the existence of a negative feedback mechanism in the OXT/OXTR system, and our results appear to support this. However, it was reported that OXT treatment reduced OXTR expression in the amygdala (71), but in this study, OXTR expression was not reduced in the amygdala. We could not exclude the possibility that the intake of a high-fat diet may have some effect on the amygdala, but this is currently unknown. Further studies on the downregulation of OXTR in the amygdala are required. In addition, it has been shown that OXT/OXTR has cell-specific effects in brain regions. OXTR neurons in the cerebral cortex are glutamate receptor neurons that control social cognition (72). In the hippocampus, OXT promotes cortical information transfer and simultaneously decreases background activity, significantly improving the signal-to-noise ratio (73). Therefore, cell-type specific study using HFD-fed mice would provide more informative insights regarding cognitive impairment.

On the other hand, administration of OXT to HFD-fed mice increased the expression of *OXT* mRNA in the hypothalamus. Moreover, in the PVN, OXT-positive cells were increased. The expression of OXT increased in the PVN. These results are consistent with the finding that central OXT administration induces hypothalamic OXT synthesis and release into the bloodstream (74). The relationship between reduced expression of OXT/OXTR signal-related molecules in the hypothalamus and changes in fear-related behavior in HFD-fed mice warrant further investigation into the complex effects of OXT/OXTR signaling on emotional and social behaviors.

OXTRs are also expressed on neurons localized in the PVN and SON, the main nuclei where OXT-expressing neurons such as the magnocellular and parvocellular neurons are also located, suggesting a positive feedback loop or activation of nearby neurons (75). Our data suggest that the positive feedback mechanism of the OXT/OXTR signaling system might display physiological roles in the response of HFD-fed mice to externally administrated OXT.

In a previous report, serum OXT was reduced in HFD-fed mice (57). To contrast, in the present study the HFD did not affect the level of serum OXT. In our experiments, we suspect that the duration of the HFD and the age of the mice might have affected the results.

This study showed that the intraperitoneal administration of 1 mg/kg of OXT to HFD-fed mice improved social and non-social cognitive functioning and increased freezing behavior. However, the effects of dose-response and the route of administration on these effects are unknown, and should be analyzed in future studies. In particular, anxiety behavior should be evaluated in a multifaceted manner utilizing multiple behavioral studies and doses.

Our study of peripherally administrated OXT to HFD-fed mice showed amelioration of non-social as well as social recognition. These results indicate the potential benefit of OXT and OXTR agonists (76, 77) in treating patients who are suffering from mental disorders induced by a HFD. OXT and artificially developed OXTR agonists have potential as a therapeutic agent to improve the cognitive decline associated with HFD.

## Data Availability Statement

All datasets generated for this study are included in the article/supplementary material.

## Ethics Statement

The animal study was reviewed and approved by Institutional Animal Care and Use Committee of Tohoku University and The Animal Experiment Committee of Nippon Flour Mills.

## Author Contributions

RH, YK, SH, SF, KNa, and KNi designed experiments for this research. RH performed the experiments and analyzed the data. RH, KNi, and YK wrote the paper.

## Conflict of Interest

RH and SF were employed by Nippon Flour Mills Co., Ltd. The remaining authors declare that the research was conducted in the absence of any commercial or financial relationships that could be construed as a potential conflict of interest.
